# Prognostic and therapeutic impact of the KRAS G12C mutation in colorectal cancer

**DOI:** 10.3389/fonc.2023.1252516

**Published:** 2023-09-15

**Authors:** Lindor Qunaj, Michael S. May, Alfred I. Neugut, Benjamin O. Herzberg

**Affiliations:** ^1^ Division of Hematology and Oncology, Department of Medicine, Columbia University, New York, NY, United States; ^2^ Herbert Irving Comprehensive Cancer Center, Vagelos College of Physicians & Surgeons, Columbia University, New York, NY, United States; ^3^ Department of Epidemiology, Mailman School of Public Health, Columbia University, New York, NY, United States

**Keywords:** KRAS G12C mutation, targeted therapies, colorectal cancer, non-small cell lung cancer, pancreatic cancer, drug development

## Abstract

*KRAS* G12C mutations are critical in the pathogenesis of multiple cancer types, including non-small cell lung (NSCLC), pancreatic ductal adenocarcinoma (PDAC), and colorectal (CRC) cancers. As such, they have increasingly become a target of novel therapies in the management of these malignancies. However, the therapeutic success of KRAS G12C inhibitors to date has been far more limited in CRC and PDAC than NSCLC. In this review, we briefly summarize the biochemistry of *KRAS* targeting and treatment resistance, highlight differences in the epidemiology of various G12C-mutated cancers, and provide an overview of the published data on KRAS G12C inhibitors for various indications. We conclude with a summary of ongoing clinical trials in G12C-mutant CRC and a discussion of future directions in the management of this disease. *KRAS* G12C mutation, targeted therapies, colorectal cancer, non-small cell lung cancer, pancreatic cancer, drug development.

## Introduction

Alterations in the membrane-associated kinase (MAPK) pathway are common in various cancers. In particular, mutations in the Kirsten rat sarcoma virus gene (*KRAS*)—first identified as a viral oncogene in Kirsten Rat sarcoma before the discovery of the human homolog—lead to constitutive activation of its associated protein (KRAS), contributing to uncontrolled cellular proliferation and survival. *KRAS* mutations are among the most common activating mutations found in tumors, frequently present in non-small cell lung cancer (NSCLC), pancreatic ductal adenocarcinoma (PDAC), endometrial carcinoma, and colorectal carcinoma (CRC).

*KRAS* mutational status in CRC is an integral part of treatment decision-making. However, direct targeting of mutant KRAS has proven exceedingly difficult. The recent development of small molecules which directly target the G12C allele of *KRAS*, has been the first successful attempt at targeting KRAS in human cancer. The KRAS G12C inhibitors sotorasib and adagrasib have demonstrated efficacy as monotherapy in *KRAS* G12C-mutant NSCLC, where roughly 15% of patients have this mutation. The G12C allele is much less common in CRC, but there is an increasing body of evidence that KRAS G12C inhibitors can be effective in CRC alone or in combination with other therapies. The rapid pace of clinical drug development in this area has already led to important clinical gains which are likely to grow.

This paper reviews the literature on *KRAS* G12C mutations, with a focus on the epidemiology and prognosis of *KRAS* G12C within CRC and the barriers that remain to the incorporation of KRAS G12C inhibitors into clinical practice in CRC.

## Biochemical background

### The MAPK pathway

The endogenous activity of the MAPK pathway begins with the binding of an extracellular mitogen to a receptor tyrosine kinase (RTK). This leads to a cascade of events which ultimately triggers transcriptional changes that result in increased cellular proliferation and survival. Common mitogen-cell surface receptor pairs which initiate this pathway include epidermal growth factor (EGF) binding to EGF receptor (EGFR) and fibroblast growth factor (FGF) binding to FGF receptor (FGFR). Binding of mitogen to RTK triggers activation of tyrosine kinase activity of the RTK cytoplasmic domain. Docking proteins then bind to phosphotyrosine residues on the activated receptor. These cell-surface signals are transduced through KRAS. KRAS exists in the cellular context in two forms, an “off” state bound to guanosine diphosphate (GDP) and an “on” state bound to guanosine triphosphate (GTP). The protein contains intrinsic GTPase activity to turn itself off, and a variety of associated proteins can shift the balance between GDP-bound (GAPs, or GTPase activating proteins) and GTP-bound (GEFs, or guanine exchange factors) KRAS. In the presence of activating signals, GEFs replace GDP with GTP, allowing KRAS to turn “on.” A downstream kinase cascade, involving serial phosphorylation of downstream kinases, such as RAF, ERK and MEK, ultimately triggers transcription factor activation, leading to increased cellular proliferation, migration and survival (1–3). In the normal cellular context, the MAPK pathway is regulated by intrinsic feedback loops and negative regulators, such as Sprouty, RAS-association domain family (RASSF) proteins, and Son of Sevenless homolog 1 (SOS-1) which help to prevent aberrant activation and signaling (4).

### Activating KRAS mutations

**Figure 1:**
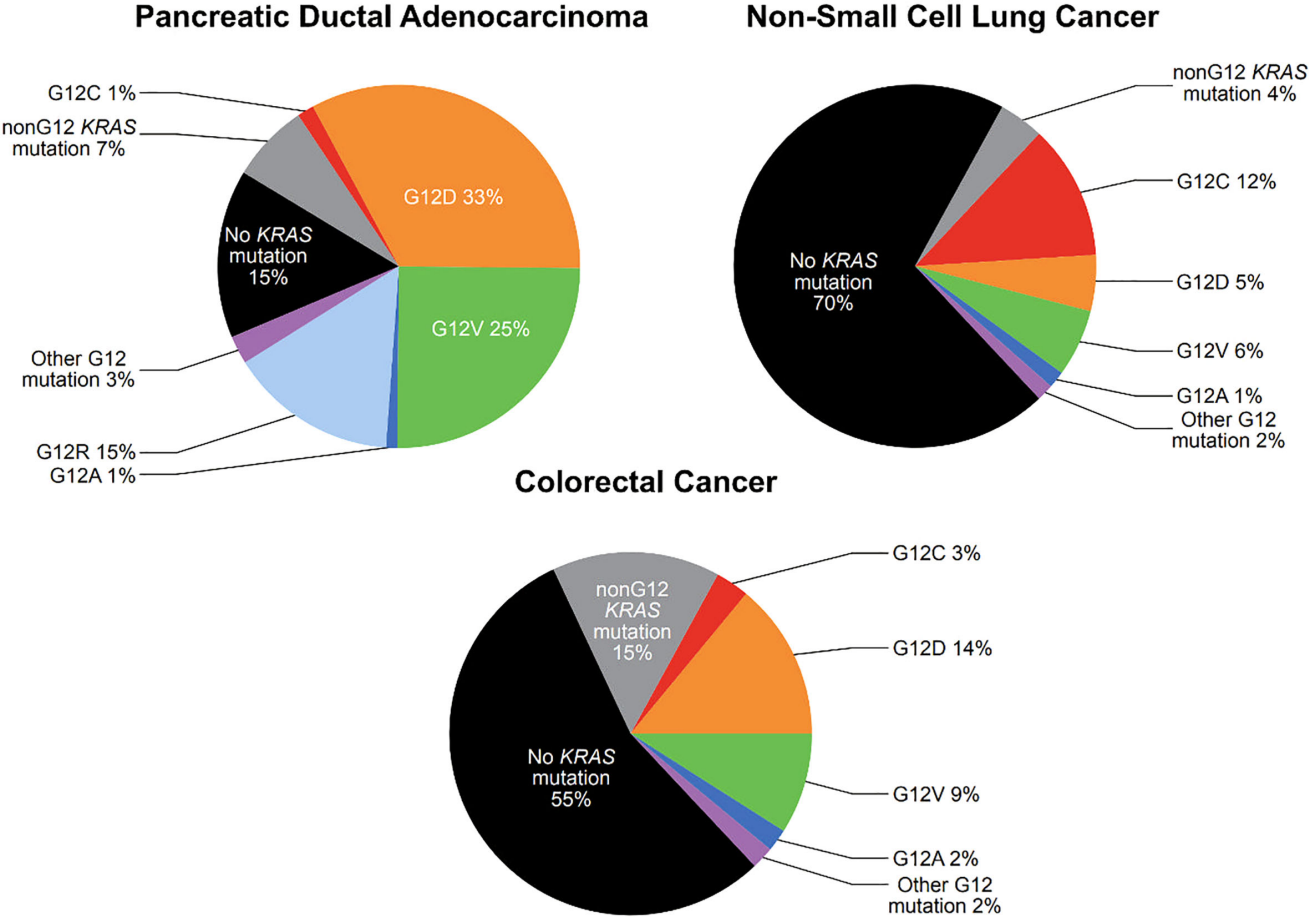
Prevalence of KRAS G12 mutations in pancreatic ductal adenocarcinoma, non-small cell lung cancer, and colorectal cancer (*see text for citations*).

There are extensive repositories documenting tumor mutational frequency in KRAS and other genes, including the Catalogue of Somatic Mutations in Cancer (COSMIC), The Cancer Genome Atlas (TCGA), and The International Cancer Genome Consortium (ICGC) (5). While the specific frequencies reported varies by source, activating mutations in *KRAS* are present in approximately 25-30% of NSCLC (6, 7), 80-95% of pancreatic ductal adenocarcinoma (PDAC) (8, 9) and 40-50% of CRC (9, 10) (**Figure 1**). The most common *KRAS* activating mutations cluster around the nucleotide-binding pocket, and involve codons 12, 13 and 61. These mutations alter the homeostatic balance between active, GTP bound KRAS and inactive, GDP-bound KRAS, either by reducing GTP hydrolysis or by increasing the rate of GTP loading. This leads to constitutive activation of the MAPK pathway (10).

### KRAS G12C mutations

Among activating *KRAS* mutations, *KRAS* G12 mutations are particularly common. *KRAS* G12 mutations are present in 25% of NSCLC, 78% of PDAC, and 30% of CRC (**Figure 1**). *KRAS* G12 mutations are missense mutations which result in the substitution of the amino acid glycine for another amino acid at codon 12, stabilizing KRAS in the active, GTP-bound state, though with slightly different features for each mutation (11). In *KRAS* G12C mutations, cysteine is substituted for glycine (12). *KRAS* G12C mutations are present in approximately 12% of NSCLC, 1.5% of PDAC, and 3% of CRC (**Figure 1**) (7, 9, 10).

## KRAS G12C mutant colorectal cancer

### Epidemiology

*KRAS* G12C mutations are present in approximately 3% of CRC cases with relatively little variation in mutation rates across geographic areas. Patient cohorts of 4,632 at MD Anderson Cancer Center in Houston, TX; 2,457 in Japan; 4,897 in Brazil; and 2,559 from a Nordic cohort with CRC who underwent genomic profiling, demonstrated *KRAS* G12C mutational rates of 2.9%, 2.8%, 3.4%, and 3%, respectively (13–15). Within the Nordic cohort, the frequency of *KRAS* G12C mutations reported at eight sites in Norway, Sweden, Finland and Denmark varied from 2%-4% (15).

*KRAS* G12C-mutant CRC appears to occur approximately equally in men and women, and has a 60% left-sided predisposition, compared with approximately 43% in unselected CRC in the US (13, 14, 16, 17). Although some studies have reported a difference in stage at presentation and metastatic sites in *KRAS* G12C-mutated CRC, the preponderance of data does not support a consistent significant difference (13–15, 18, 19).

### Prognosis

Although there is some variability in studies, most large retrospective studies have demonstrated an association between *KRAS* G12C mutations and worse progression-free survival (PFS) and overall survival (OS) when compared with other *KRAS*-mutated CRC (13, 14, 19, 20). *KRAS* G12V mutations may also convey a poor prognosis (19, 20). One study, which included 187 patients with *KRAS* G12C mutations and *de novo* metastatic disease, demonstrated a median PFS of 6.4 months, 3.9 months, and 3.0 months after first, second and third lines of therapy, with an OS of 25.1 months (14).

### Genetic and molecular profile

The most common mutations in CRC occur in *APC*, *TP53*, *KRAS* and *PIK3CA (* 21, 22 *).* Rates of *APC* and *TP53* mutations do not vary consistently based on the presence or type of *KRAS* mutation (14, 19). *PIK3CA* mutations are less common in patients with *KRAS* G12C mutations than in patients with *KRAS* mutations at alternative sites (14, 19). Mutations in other genes involved in the MAPK pathway, such as *BRAF* and *NRAS*, are much less likely to be mutated in CRC cases with *KRAS* mutations including G12C mutations (14, 17, 19). This is consistent with molecular data from other cancers, such as NSCLC, in which *KRAS* mutations are almost always exclusive of other strong driver mutations, such as EGFR (23, 24).

Alterations in the microsatellite instability (MSI) pathway are of particular clinical interest as they facilitate sensitivity to immunotherapy (25, 26). MSI is caused by a deficiency of mismatch repair (dMMR) and is found in approximately 10-15% of CRC. Rates of dMMR are very low in *KRAS* G12C-mutant CRC (1.6% vs 2.9% in other *KRAS* mutants and 4.5% in all cases) (19).

*KRAS* G12C mutated CRC displays lower rates of CIMP pathway activation (hypermethylation) compared with *KRAS* WT CRC (14). Overall, these data point to *KRAS* G12C CRC as being a biologically distinct class of CRC with different therapeutic opportunities.

## Targeting KRAS

### Failure of mutational nonspecific targeting

Given the high frequency of *KRAS* mutations across multiple tumor types, including approximately 45% of CRC, mutant KRAS has been a pharmacologic target for over three decades (27). Direct KRAS targeting has been extremely challenging, as its affinity for GTP and mostly featureless surface precluded traditional approaches of small-molecule medicinal chemistry (28). This led to a variety of secondary approaches to RAS targeting, either by attempting to target other pathway “nodes” or the translation and processing of KRAS on its way to the cell surface. The enzyme farnesyltransferase, for example, catalyzes the addition of a farnesyl lipid group to a carboxyl terminal cysteine of KRAS, enabling localization to the cell membrane (29). Farnesyltransferase inhibitors were consequently an early class of pan-*RAS* inhibitors which, despite many preclinical successes, failed to show success in the treatment of *KRAS*-mutant cancers, possibly due to alternative prenylation of KRAS (30, 31).

In the context of repeated failures to directly target KRAS, it is notable that other inhibitors of the MAPK pathway have proven central in defining treatment paradigms for CRC. The *EGFR* inhibitors cetuximab and panitumumab proved effective in *RAS* WT CRC (32, 33) and the *BRAF* inhibitor encorafenib demonstrated efficacy in the treatment of *BRAF* V600E mutant CRC (34). Confining EGFR inhibitors, which act “upstream” of KRAS, to only KRAS WT disease makes conceptual sense, but limited the usefulness of these drugs.

### Targeting KRAS G12C

The first success in directly targeting KRAS grew out of the unique biochemistry of the codon 12 glycine-to-cysteine (G12C) mutation. Cysteine thiols are uniquely nucleophilic amongst amino acid side chains. This is classically seen in formation of disulfide bonds and made the cysteine residue in mutant *KRAS* G12C an attractive pharmacologic target (12). This property enabled disulfide-fragment-based techniques to screen hundreds of potential small-molecule targets of G12C-mutant *KRAS* in a mutant-specific manner, sparing WT *KRAS*. Building on the fact that G12C was targetable, two clinical compounds have now obtained approval: sotorasib and adagrasib—with a half-dozen or more molecules close behind.

### Targeting KRAS G12C in lung cancer

Sotorasib was the first *KRAS* G12C inhibitor approved in the United States. It demonstrated efficacy and acceptable toxicity in patients with previously treated *KRAS* G12C mutant advanced NSCLC. In CodeBreaK100, a single-arm phase 2 trial of 126 patients, Sotorasib demonstrated an objective response rate (ORR) of 37% with a median duration of response of 11.1 months. PFS was 6.8 months and OS was 12.5 months (35). A subsequent randomized controlled trial of 345 patients demonstrated superior PFS with sotorasib than with docetaxel (5.6 vs 4.5 months; HR 0.66 with p=0.0017) and fewer grade 3 or worse adverse events (AEs) (36). Common treatment-related AEs seen with sotorasib include diarrhea, nausea, vomiting, fatigue and liver function test elevation (35, 36).

Adagrasib was evaluated in KRYSTAL-1, a phase 2 study of 116 patients with previously treated *KRAS* G12C mutant NSCLC. Adagrasib demonstrated similar outcomes to sotorasib in this cohort, with an ORR of 43%, median duration of response of 8.5 months, disease control rate of 80%, PFS of 6.5 months and OS of 12.6 months. Common AEs were similar (37). These two proof-of-principle trials for direct KRAS inhibition were landmarks that led to the exploration of new KRAS-targeted drugs and *KRAS* G12C strategies in other histologies.

### Targeting KRAS G12C in pancreatic cancer

Sotorasib was evaluated in a phase 1-2 trial of 38 patients with previously treated metastatic *KRAS* G12C-mutant PDAC, with a median of two prior lines of prior therapy. The ORR was 21%; PFS was 4.0 months and OS was 6.9 months (38)

These promising but time-limited responses have prompted further investigation into the mechanisms of KRAS G12C inhibitor resistance, and drug combinations which could help increase response rates or prolong the duration of response (39).

### Targeting KRAS G12C in colorectal cancer

Early clinical studies of KRAS-targeted therapies in G12C-mutant colorectal cancer showed less activity than what is reported in NSCLC. A single-arm study of sotorasib monotherapy in previously treated *KRAS* G12C-mutant CRC, for instance, demonstrated an overall response rate of 9.7% across 62 evaluable patients (40). More recently, adagrasib alone was tested in a cohort of 43 CRC patients in the KRYSTAL-1 trial. The investigator-assessed objective response rate was 19%, with a median PFS of 5.6 months (4.1-8.3, 95%CI) and median OS of 19.8 months (12.5-23.0, 95%CI) (41). Safety data was comparable to the results seen in NSCLC, with the most common adverse events being diarrhea, nausea, vomiting, and fatigue (all occurring in >40% of patients).

The mechanisms underlying these discordant responses to G12C targeting in CRC and NSCLC are not clearly understood. Why do a smaller percentage of patients respond? One hypothesis is that CRC tumors undergo a significant rebound in ERK phosphorylation soon after starting KRAS inhibition, suggesting rapid development of treatment-related adaptive signaling resistance (42). *KRAS*-mutant CRC has also been shown to have higher levels of “upstream” receptor tyrosine kinase phosphorylation as compared to NSCLC, especially in EGFR. EGFR-mediated resistance to other targeted therapies has been seen in CRC, including with BRAF inhibition, making this a natural place to look (43).

This observation has prompted studies combining small molecule KRAS inhibitors with anti-EGFR monoclonal antibodies in CRC. CodeBreaK-101, for example, included a cohort of 40 metastatic CRC patients treated with a combination of sotorasib and panitumumab, noting an ORR of 30% (16.6-46.5, 95%CI) (44). Adagrasib was similarly evaluated with or without concurrent cetuximab in 28 heavily pretreated G12C-mutant CRC patients. Without cetuximab, ORR was 19%; with cetuximab, the ORR was 46%. Notably, the standard paradigm in CRC has been that *KRAS*-mutant tumors do not have responses to anti-EGFR mAb therapy. Similar results have been presented for compounds earlier in development, including GDC-6036, a G12C inhibitor being developed by Genentech, which showed an even higher ORR of 66% when combined with cetuximab (45). Overall these results suggest that this combination may receive approval concurrently, or before, monotherapy treatment for *KRAS* G12C CRC. Still, despite the improved responses relative to KRAS inhibition alone, these results suggest a similar discrepancy in efficacy between CRC and NSCLC patients receiving identical combinations. These trials are summarized in **Table 1**.

**Table 1:** Summary of key results from KRAS G12C inhibitor trials in CRC.

Trial	Intervention	Number of Patients	Treatment Setting	ORR (95% CI)	Median PFS (95% CI)
CodeBreak-100	Sotorasib	62	2^nd^ line	9.7% (3.6-19.9)	Not reported
CodeBreaK-101	Sotorasib + panitumumab	40		30% (16.6-46.5)	Not reported
KRYSTAL-1	Adagrasib	43	Heavily pretreated	19% (8-33)	5.6 months (4.1-8.3)
	Adagrasib + cetuximab	28		46% (28-66)	6.9 months (5.4-8.1)
KRYSTAL-7	Adagrasib + pembrolizumab	53	1^st^ line	49% (35-63)	Not reported

### The KRAS G12C development pipeline

These somewhat blunted responses in CRC have not deterred continued development of new G12C inhibitors, either as monotherapy or in novel combinations for this indication. Using a search term of “G12C” for registered clinical trials yielded 76 unique entries (last updated April 27, 2023), of which 39 were trials that included patients with G12C-mutant CRC. Thirty-three studies were excluded from further review because they included only lung cancer patients, two were excluded because they were diagnostic/non-interventional studies, and one each was excluded for not being specific to G12C-mutant tumors or having *KRAS* mutations as an exclusion criterion. Within these exclusions, there are 12 distinct G12C inhibitors currently under investigation (**Table 2**).

**Table 2:** KRAS G12C inhibitors currently in clinical development based on registered trials publicly listed on ClinicalTrials.gov.

Drug	Sponsor
D3S-001	D3 Bio
GFH925 (IBI351)	Genfleet Therapeutics
JAB-21822	Jacobio Pharmaceuticals
JNJ-74699157	Janssen
YL-15293	Yingli Pharma
HBI-2438	Huyabio
JDQ443	Novartis
GDC-6036	Genentech
LY3537982	Eli Lilly
MK-1084	Merck
HS-10370	Jiangsu Hansoh
BPI-421286	Betta Pharmaceuticals

Essentially all are KRAS-GDP covalent inhibitors. Subtle differences in pharmacology and pharmacokinetics between these compounds might translate into efficacy benefits or differing potential in combination therapy regimens. Novartis’ JDQ443, for example, binds without involving the H95 residue (46). As a result of its H95-independent mechanism, it appears to maintain activity even among tumors with G12C/H95 double *KRAS* mutations, which might help mitigate some acquired resistance (47). Such second-site mutations in H95 appear to occasionally mediate resistance to other KRAS G12C inhibitors, such as adagrasib (48).

Janssen’s JNJ-74699157 similarly binds near the switch II pocket through an interaction with a differing cysteine residue, suggesting it may be able to evade these resistance mechanisms as well. In a phase 1 study of 10 patients across multiple tumor types, however, zero patients had an objective response and six experienced dose-limiting muscle toxicity in the form of CPK elevations (49). In contrast, the KontRASt-01 trial of JDQ443 is ongoing, with no alarming safety signals publicly reported to date (50).

Most recently, Eli Lilly’s LY3537982 presented a preliminary read-out of its phase 1 LOXO-RAS-20001 study at the 2023 AACR Annual Meeting (51). The ORR specifically among patients with CRC (n=20) receiving LY3537982 as monotherapy was 10%, compared to 42% among those with pancreatic (n=12) or 38% in NSCLC not previously treated with a KRAS G12C inhibitor (n=8). In one cohort of 11 CRC patients, the sponsors investigated the novel agent in combination with cetuximab, demonstrating an ORR of 45%. Liver enzyme elevations were more common in this cohort. This trial was notable for including several patients who had stopped prior G12C inhibitors for toxicity. Whether that toxicity would have abated on its own or LY3537982 has a more tolerable safety profile remains to be determined in larger studies.

### Seeking synergies with other targets

Apart from these investigations into novel monotherapies, there is considerable interest in combining *KRAS* G12C inhibitors with other targets as a way to either potentiate their clinical effect or counteract the development of resistance. While the addition of anti-EGFR antibodies was one of the earliest studied combinations, concurrently targeting BRAF, MEK, and other downstream steps in the RAS/MAPK signaling pathway holds theoretical promise as well (52). *In vitro* and mouse models of lung cancer, for instance, have shown that adding the mTOR inhibitor everolimus to a KRAS G12C inhibitor reduces tumor cell viability (53). Dozens of other preclinical investigations have supported combination therapies.

One prominent combination is with checkpoint inhibitors, based on the hypothesis that KRAS inhibition induces pro-inflammatory changes in the tumor microenvironment of KRAS mutated tumors. Early mouse studies combining sotorasib and anti-PD-1 therapy illustrated increased CD8+ T-cell infiltration into the tumor microenvironment, an effect not seen in subjects treated with checkpoint inhibition alone (54). This finding correlated with improved *in vivo* efficacy, with 9/10 mice achieving complete responses, compared to 1/10 in the monotherapy arms. Such responses in preclinical studies are typically not seen with chemotherapy-immunotherapy combinations.

In an attempt to replicate that success in humans, the KRYSTAL-7 trial tested the combination of adagrasib and pembrolizumab as a first-line therapy in patients with advanced *KRAS* G12C-mutant NSCLC. The preliminary results presented at the 2022 ESMO Immuno-Oncology Annual Congress featured an objective response rate of 49% (35-63, 95%CI) across 53 patients, with rates at least directionally varying by PD-L1 tumor proportion score (TPS): 59% for TPS≤50, 48% for TPS 1-49, and 30% for TPS<1 (55). Sotorasib has similarly reported results for a combination arm with pembrolizumab, but this combination led to a notably high rate of liver toxicity. The main current question is whether this liver toxicity will be a class effect or if it will be specific only to some KRAS G12C inhibitors. Trials are likely to answer this question in the coming months and years.

Several ongoing trials include combinations of existing RAS inhibitors with SHP-2 inhibitors, including Novartis’s TNO 155, BridgeBio’s BBP-398, and Revolution Medicine’s RMC-4630. A combination of BBP-398 and sotorasib had a synergistic inhibitory effect on MAPK signaling and cell viability in an *in vitro* model and was granted fast-track designation (56). SHP-2 is a non-receptor type protein tyrosine phosphatase encoded by the gene PTPN11. Because SHP-2 acts downstream of most receptor tyrosine kinases and is essential for RAS activation and signaling rebound, there is a preclinical rationale to support combination therapy. Monotherapy trials of SHP-2 inhibition have shown modest efficacy in *KRAS*-mutated tumors, especially *KRAS* G12C (57), but most compounds are now in development in combination with other MAP/ERK pathway inhibitors, such as the KRAS G12C inhibitors. Sotorasib combinations have started to read out (58), and further data will clarify whether this combination has value in CRC or other cancers.

There are novel molecules targeting other companion proteins of RAS for which combination therapy hypotheses are natural. One is BI-1701963, a pan-RAS inhibitor targeting the catalytic site of SOS-1. SOS-1 is a GEF that binds to GDP-bound RAS proteins and is critical for tumorigenesis; in fact, the absence of SOS-1 has been to shown to have a deleterious effect on cancer cells containing *KRAS* mutations (59, 60). In a phase 1 dose-escalation study of 31 patients treated with BI-1701963 monotherapy, none achieved clinical responses and only seven had at least stable disease (61).

## Discussion

*KRAS* is one of the most common and most difficult to target. The advances which led to direct KRAS G12C inhibition combined new pharmacology, chemistry, and thirty years of basic biology investigating RAS biochemistry, structural biology, and signaling adaption. The short eight years from the first disclosure of a covalent KRAS G12C binder to the demonstration of clinical efficacy and obtaining accelerated FDA approval in NSCLC is remarkable. While somewhat behind the NSCLC development, it is likely that KRAS G12C inhibitors will also receive FDA approval in CRC in the coming years.

But the efficacy for KRAS G12C inhibitors observed so far in CRC is its own evolving story that differs from other tumor types. Monotherapy response rates remain low, and it is plausible that combination therapies, such as with EGFR mAbs, will be the first approved regimens. More broadly, the duration of response for most of these molecules is relatively short.

In addition to combinations, next-generation molecules such as KRAS-GTP inhibitors may hold particular promise to increase response rates and perhaps tame some signaling-based resistance mechanisms (62). Direct pan-RAS or pan-KRAS inhibitors, which inhibit RAS regardless of mutated allele (and include on-target WT inhibition), have also been described, with lots of preclinical data and early hints of clinical efficacy (63, 64). It is conceivable that both primary and acquired resistance emergence, and resistance patterns— along with clinical efficacy—will be different for these molecules. It is also possible that toxicity will limit dosing in a clinically meaningful way, but data has yet to be shown.

There remains a great need for KRAS-directed therapy in CRC for both G12C and other alleles. With proof-of-concept activity now firmly in hand, it is likely that these therapies will become a significant part of standard CRC therapy for patients with qualifying molecular alterations in the not-distant future. The hope is that advances in patient survival, reduced progression, and perhaps even cure rates in the adjuvant setting will follow. For now, we are very much at the beginning of this frontier, rather than the end.

## Author contributions

AN and BH conceived the study and initiated the report. LQ and MM reviewed the literature and prepared the tables and figures and did the review. All four authors contributed portions of the initial draft and all four authors reviewed and edited the manuscript and all the authors approved the final manuscript. All authors contributed to the article and approved the submitted version.
